# Retrograde robot-assisted radical prostatectomy in a patient with localized prostate cancer: a case report

**DOI:** 10.3389/fsurg.2026.1880320

**Published:** 2026-08-12

**Authors:** Zdravka Harizanova, Ferihan Ahmed-Popova, Vasil Pavlov, Elena Bozhikova

**Affiliations:** 1 Department of Anatomy, Histology and Embryology, Faculty of Medicine, Medical University – Plovdiv, Plovdiv, Bulgaria; 2 Urology Department, UMBAL “Kaspela”, Medical University – Plovdiv, Plovdiv, Bulgaria; 3 Department of Biomedical Sciences, Mercer University School of Medicine, Columbus, GA, United States

**Keywords:** case report, prostate, prostatic carcinoma, radical prostatectomy, retrograde nerve-sparing, robot-assisted radical prostatectomy

## Abstract

**Introduction:**

Prostate cancer represents one of the most prevalent malignancies affecting the male population worldwide. With increasing survival rates, greater attention has been directed toward postoperative complications, particularly erectile dysfunction and urinary incontinence.

**Patient concerns:**

A 70-year-old male patient with histologically confirmed prostate carcinoma reported lower urinary tract symptoms including dysuria and nocturia.

**Diagnosis:**

The pathological diagnosis was organ-confined acinar adenocarcinoma of the prostate, pT2, ISUP Grade Group 1, with negative surgical margins (R0).

**Interventions and outcomes:**

After pelvic ultrasound, consultation with an anesthesiologist and a cardiologist, the patient underwent retrograde robot-assisted radical prostatectomy.

**Conclusion:**

This case illustrates the practical application of the retrograde approach, emphasizing its anatomical rationale and technical considerations rather than demonstrating superiority over conventional antegrade nerve sparing. Careful patient selection, sound anatomical knowledge, and adherence to oncological principles remain essential for balancing cancer control with functional preservation. Further prospective comparative studies are required to clarify the role of retrograde nerve-sparing dissection in contemporary robot-assisted radical prostatectomy.

## 1 Introduction

Prostate cancer represents one of the most prevalent malignancies affecting the male population worldwide. Robot-assisted radical prostatectomy (RARP) has become a standard therapeutic option for patients with localized prostate cancer and is associated with excellent oncological outcomes, with reported 5-year survival rates of approximately 95% in appropriately selected patients (1). The widespread implementation of prostate-specific antigen (PSA) screening has contributed to earlier diagnosis and stage migration, resulting in an increasing number of patients presenting with low-volume and low-stage disease (2). Consequently, as oncological outcomes and survival rates have improved, increasing attention has been directed toward postoperative functional outcomes, particularly erectile dysfunction and urinary incontinence, which significantly affect patients' quality of life, psychosocial well-being, and intimate relationships (3, 4).

The surgical management of prostate cancer has undergone substantial evolution since the first radical prostatectomy described by Hugh Young in 1904 using the transperineal approach (5), followed by Millin's introduction of the retropubic open radical prostatectomy in 1947 (6). Although these techniques represented important milestones in prostate cancer surgery, they were associated with considerable morbidity, including significant blood loss, urinary incontinence, and impaired sexual function. Subsequent technological advances led to the development of minimally invasive approaches, including laparoscopic prostatectomy, first reported by Schuessler et al. in 1997 (7), and robot-assisted laparoscopic prostatectomy, initially described by Abbou et al. in 2000 (8).

When evaluating surgical modifications in radical prostatectomy, the primary considerations remain oncological safety, perioperative morbidity, and preservation of long-term functional outcomes. Robot-assisted surgery provides enhanced three-dimensional visualization, improved instrument articulation, and improved control of intraoperative bleeding, contributing to reduced estimated blood loss, postoperative hemoglobin reduction, shorter hospital stays, and favorable functional outcomes compared with conventional approaches (9). Furthermore, several studies have demonstrated advantages of RARP regarding postoperative urinary and sexual function, supporting its role as a preferred minimally invasive approach for selected patients with localized prostate cancer (10).

Despite these advances, preservation of urinary continence and erectile function remains one of the major challenges of radical prostatectomy. These functional outcomes depend largely on meticulous preservation of neurovascular bundles (NVBs) and surrounding anatomical structures. Therefore, optimization of nerve-sparing strategies remains an important objective in modern prostate cancer surgery.

The conventional nerve-sparing technique during robot-assisted radical prostatectomy is most commonly performed using an antegrade approach, in which the prostatic pedicles are controlled first, followed by progressive release of the NVB from the prostate base toward the apex. Although this technique is widely established and reproducible, early manipulation of vascular structures and surrounding tissues may theoretically expose the NVB to traction or thermal injury before its anatomical course is completely defined.

The retrograde nerve-sparing approach represents an alternative sequence of dissection in which the NVB is initially identified at the level of the prostatic apex and subsequently released in a distal-to-proximal direction. This strategy allows the anatomical course of the neurovascular structures to be established before proceeding toward the prostatic base and vascular pedicles, potentially facilitating more controlled preservation of the NVB. Importantly, the retrograde approach does not represent a newly introduced surgical technique, but rather an alternative dissection strategy that may provide specific anatomical advantages in selected patients undergoing nerve-sparing robot-assisted radical prostatectomy. Although the retrograde nerve-sparing approach has been described previously, detailed contemporary case-based reports illustrating its stepwise robotic execution, anatomical rationale, and practical technical considerations remain relatively limited. Such reports may provide educational value for surgeons seeking to optimize nerve preservation while adhering to established oncological principles.

The present case report does not introduce a novel surgical technique or seek to demonstrate superiority of the retrograde approach over the conventional antegrade dissection. Instead, it describes the practical application of a retrograde nerve-sparing strategy during robot-assisted radical prostatectomy, emphasizing the anatomical rationale, key technical steps, and intraoperative decision-making. By providing an illustration of this alternative dissection sequence, this report aims to contribute to the educational literature on nerve-sparing robot-assisted radical prostatectomy.

## 2 Case description

A 70-year-old male patient with histologically confirmed prostate carcinoma was hospitalized for surgical treatment. The patient reported lower urinary tract symptoms including dysuria and nocturia. His past medical history was significant for left-sided nephrectomy performed in 2015, laparoscopic cholecystectomy in 2024, and inguinal hernioplasty in 2025. The patient also had a history of type 2 diabetes mellitus and arterial hypertension, but his chronic medications and the indication for previous nephrectomy were not available in the reviewed medical records. The patient had no familial background of prostate carcinoma. On admission, the patient was in good general condition, conscious, oriented, and afebrile. The skin and visible mucous membranes were pale pink. The tongue was moist and clean. The head and neck had normal configuration, and peripheral lymph nodes were not palpably enlarged. The thorax was symmetric both statically and dynamically, with bilateral vesicular breath sounds and no wheezing or crepitations. Cardiac examination revealed regular rhythm with a heart rate of 80 beats per minute, clear heart sounds, and no pathological sounds. Blood pressure was 120/80 mmHg. The abdomen was soft, non-tender, and respiratory-mobile on palpation. Surgical scars were present in the right inguinal region, left hypogastrium, and right abdominal quadrant.

Laboratory investigations revealed the following results: WBC 9.32 × 10⁹/L, RBC 5.68 × 10¹²/L, hemoglobin 166 g/L, hematocrit 0.49, MCV 86.3 fL, MCH 29.2 pg, and ESR 10 mm/h, PSA 12 ng/mL. Coagulation parameters were within normal limits, including bleeding time 210 s, clotting time 420 s, and prothrombin index 105%. Serum urea was 7.6 mmol/L and creatinine 146 µmol/L. Total serum protein was 77 g/L and albumin 45 g/L.

Urinalysis showed pH 5.0, proteinuria (1+), and glycosuria (4+), with negative ketone bodies and bilirubin, and normal urobilinogen levels.

Ultrasonographic examination demonstrated a right kidney with normal size, position, and preserved parenchyma without hydronephrosis. The left kidney was absent, consistent with the previous nephrectomy. The urinary bladder had smooth walls with well-defined contours. The prostate gland appeared heterogeneous with dimensions of 63.1 × 58.7 × 49.4 mm.

Detailed biopsy parameters, including the biopsy approach, number of cores, percentage of tumor involvement, and ISUP Grade Group, as well as preoperative multiparametric MRI findings and PI-RADS classification, were not available in the reviewed medical documentation. The diagnosis of prostate carcinoma was established before hospital admission based on histopathological confirmation, and the patient was referred for definitive surgical treatment.

Based on the histopathologically confirmed diagnosis of prostate carcinoma, together with the clinical, laboratory, and imaging findings, the patient was scheduled for robot-assisted radical prostatectomy under general inhalational anesthesia.

After induction of general anesthesia and placement of the patient in a steep Trendelenburg position, the patient was secured to the operating table to prevent sliding during robotic manipulation. Following sterile preparation and draping of the abdomen and perineum, pneumoperitoneum was established using a Veress needle technique. The initial camera port was introduced under direct laparoscopic visualization after safe entry into the peritoneal cavity. Additional robotic and assistant ports were subsequently placed under direct vision according to the standard configuration for the da Vinci X surgical platform. The da Vinci X robotic system was docked from the patient's side, and robotic instruments were introduced, including a 30° endoscope, fenestrated bipolar forceps, monopolar curved scissors, and ProGrasp forceps. The procedure was performed as a robotic-assisted radical prostatectomy (RARP) using a bilateral nerve-sparing approach. An interfascial dissection was performed on the left side and an intrafascial dissection on the right, with the choice of dissection plane tailored according to intraoperative anatomical findings and the surgeon's intraoperative judgment.

### 2.1 Exposure and anterior dissection

**Figure 1:**
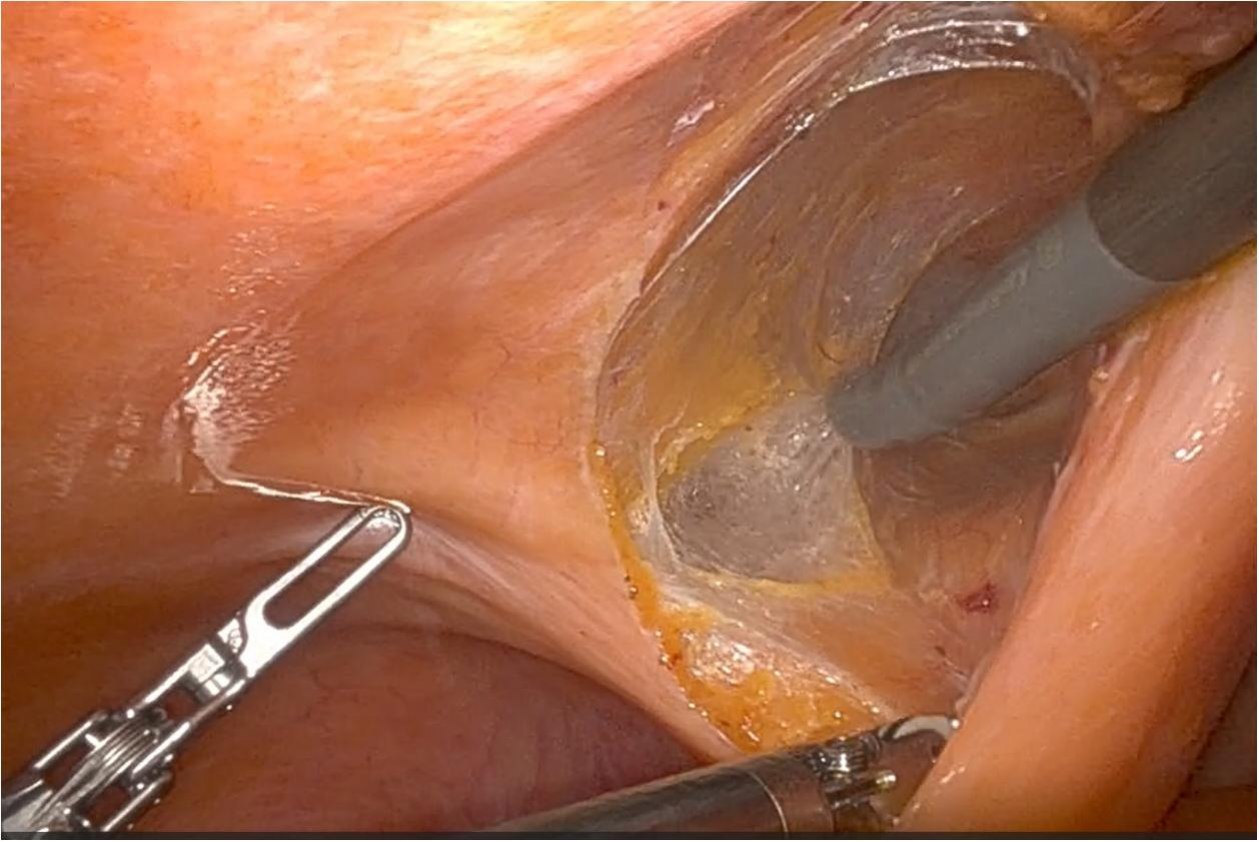
Incision of the pelvic fascia during the initial stage of robot-assisted radical prostatectomy to expose the lateral aspect of the prostate before nerve-sparing dissection.

The peritoneum was opened, and the Retzius space was developed. The perivesical adipose tissue was removed to expose the endopelvic fascia, puboprostatic ligaments, and dorsal venous complex (DVC). The endopelvic fascia was incised bilaterally, allowing mobilization of the prostate and facilitating identification of the periprostatic fascial planes. The DVC was controlled using a running suture ligation technique, followed by careful division of the venous complex. Hemostasis was verified before proceeding with bladder neck dissection (Figure 1).

### 2.2 Bladder neck dissection and posterior plane development

The bladder neck was dissected circumferentially, preserving bladder neck length whenever oncologically appropriate. The prostate was separated from the bladder, allowing identification of the seminal vesicles and bilateral vas deferens. The vas deferens were dissected, clipped, and divided, followed by mobilization of the seminal vesicles with careful preservation of surrounding tissue. Posterior dissection was then initiated by developing the plane between Denonvilliers' fascia and the anterior rectal wall. The dissection proceeded toward the prostatic apex under continuous visualization of the rectal plane to avoid injury.

### 2.3 Retrograde neurovascular bundle preservation

**Figure 2:**
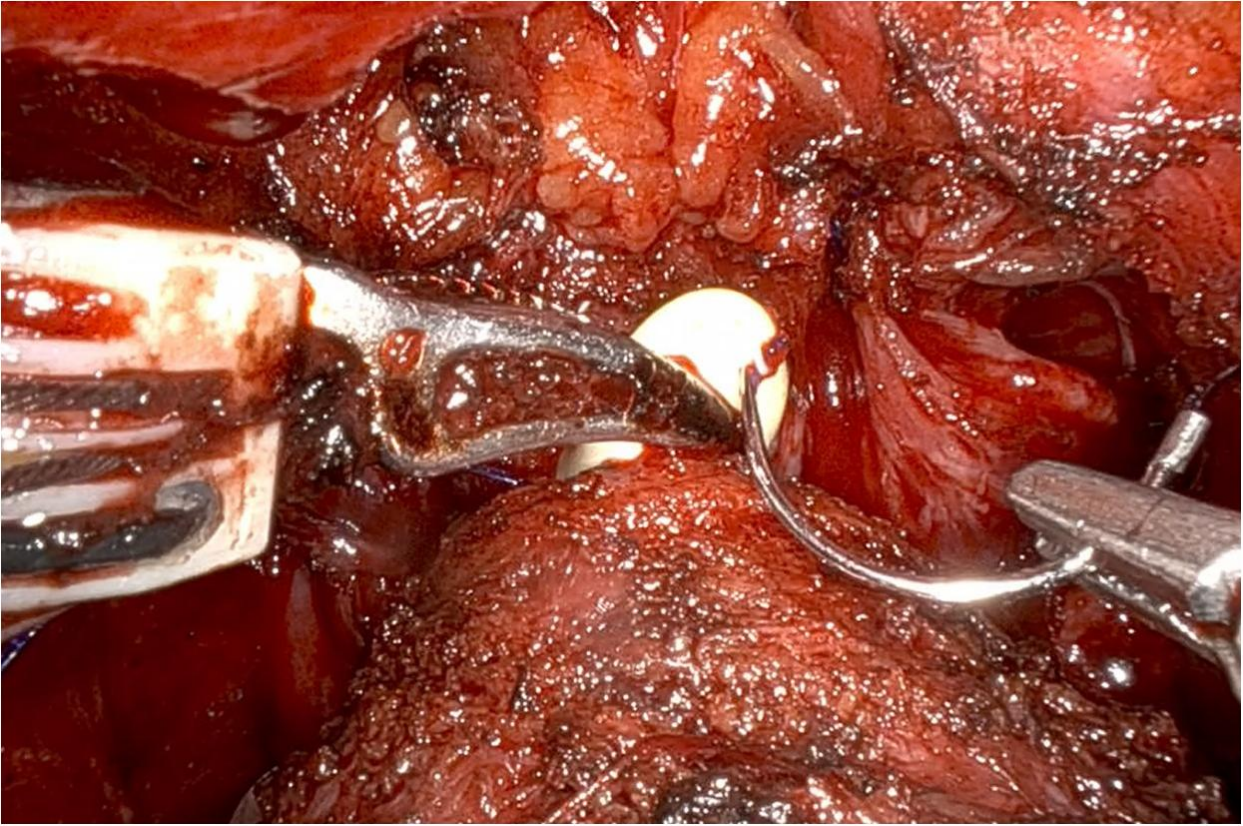
Intraoperative view illustrating the retrograde nerve-sparing dissection during separation of the neurovascular bundle from the prostate.

A retrograde bilateral NVB-sparing technique was performed beginning at the apical region and progressing toward the base. The lateral prostatic pedicles were dissected sharply, with bilateral preservation of the neurovascular bundles. An interfascial dissection plane was used on the left side, whereas an intrafascial plane was used on the right, according to intraoperative assessment of oncological safety and anatomical findings. The vascular pedicles were controlled using clips and divided sharply to minimize thermal spread and avoid injury to adjacent neural structures. No energy-based sealing devices were used in close proximity to the NVBs (Figure 2).

### 2.4 Apical dissection and specimen removal

At the prostatic apex, meticulous dissection was performed to preserve the maximum functional length of the membranous urethra while ensuring oncological safety. Following complete apical dissection, the membranous urethra was transected, completing the retrograde neurovascular bundle-sparing procedure. The prostate, seminal vesicles, and vas deferens were removed *en bloc* and placed into an extraction bag.

### 2.5 Vesicourethral reconstruction

**Figure 3:**
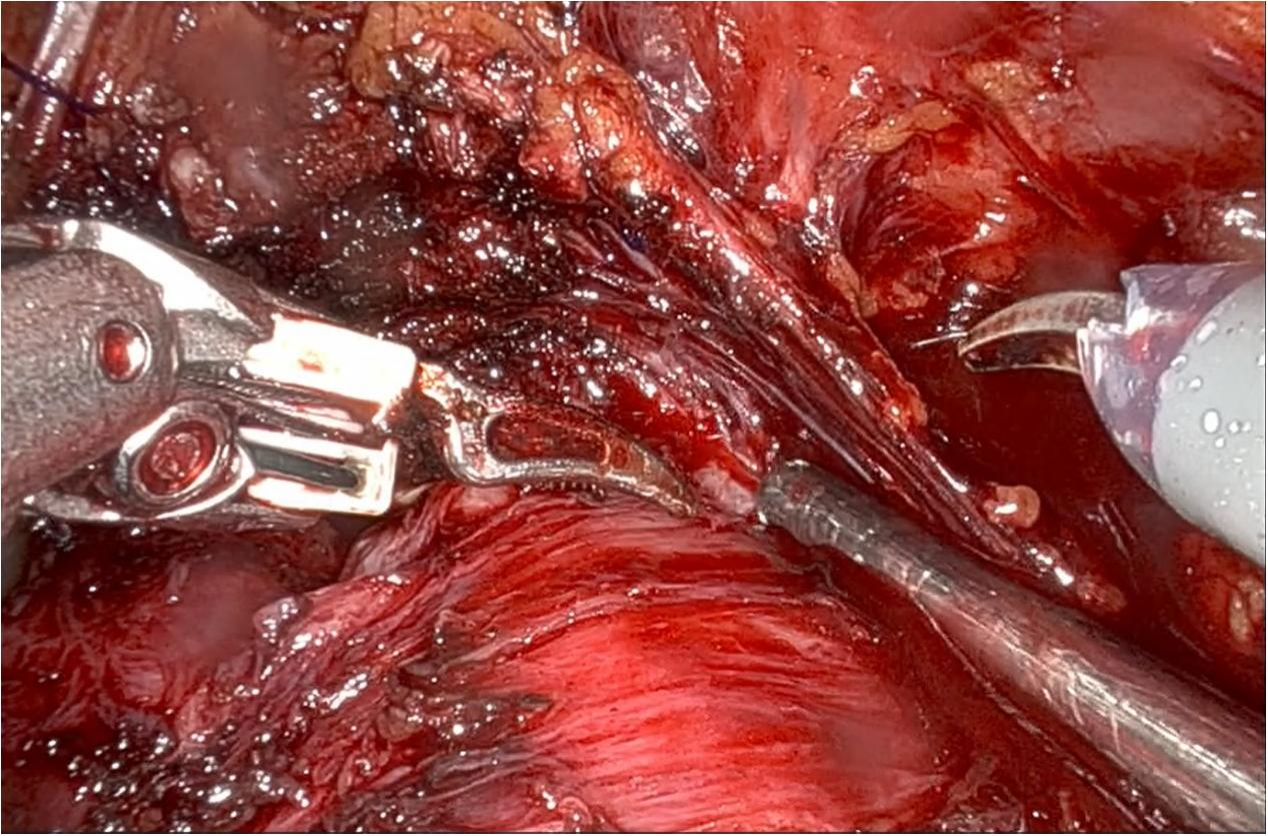
Vesicourethral anastomosis performed after completion of prostate removal, restoring continuity between the bladder neck and the urethra.

A posterior reconstruction was performed prior to the vesicourethral anastomosis. Vesicourethral anastomosis was completed using two continuous barbed sutures in a running fashion over an 18 Fr Foley catheter. The anastomosis was tested for watertight integrity by bladder filling with sterile saline (Figure 3).

### 2.6 Completion of the procedure

The specimen was placed into an endoscopic retrieval bag and removed through the enlarged assistant port site. Pneumoperitoneum was released, all ports were removed under direct visualization, and fascial defects were closed with interrupted sutures. Skin incisions were closed using standard techniques, and sterile dressings were applied. The surgical specimen was submitted for histopathological evaluation.

### 2.7 Perioperative outcomes

• Total operative time: 200 min

• Console time: 150 min

• Estimated blood loss: 150 mL

• Intraoperative complications: None

• Conversion to open surgery: No

• Intraoperative transfusion: No

## 3 Тable showcasing a timeline with relevant data from the episode of care

To facilitate the presentation of the patient's clinical course, the major events throughout the episode of care are summarized chronologically in Table 1. The table provides an overview of the key diagnostic, therapeutic, and postoperative milestones (Table 1).

**Table 1:** Timeline table with relevant data from the episode of care.

Time Point	Clinical Stage	Key Findings/Indication	Surgical/Clinical Intervention	Outcome and Patient Status
Day 0	Admission	Symptoms leading to hospital presentation	Initial clinical and laboratory assessment	Hemodynamically stable; candidate for surgery
Preoperative Period	Diagnostic Workup	Prostate biopsy confirmed adenocarcinoma	Preoperative planning with focus on access technique	Patient optimized for surgery
Day 1	Surgical Intervention	Indication maintained	Application of the selected surgical access technique	Procedure completed successfully; no intraoperative complications
Postoperative Day 1	Early Postoperative Phase	Pain and recovery assessment	Analgesia and monitoring	Mild postoperative pain; stable condition
Postoperative Days 2–4	Recovery Phase	Gradual functional improvement	Mobilization and routine postoperative care	No complications; progressive recovery
Discharge Day 5	Hospital Discharge	Clinically stable	Final evaluation	Discharged in good general condition

## 4 Diagnostic assessment

Histopathological examination of the prostate specimen revealed a moderately differentiated prostatic adenocarcinoma with a Gleason score of 3 + 3 = 6 (ISUP Grade Group 1). No tumor infiltration of the fibrous prostatic capsule or periprostatic tissues was identified. Perineural invasion was not observed. The seminal vesicles and vas deferens were free of tumor infiltration. The capsule adjacent to the left neurovascular bundle showed no evidence of tumor involvement. Similarly, the capsular margin at the prostatic apex was free of malignant infiltration. A mild lymphoid stromal reaction was present.

The final pathological diagnosis was organ-confined acinar adenocarcinoma of the prostate, Gleason score 3 + 3 = 6 (ISUP Grade Group 1), staged as pT2, with negative surgical margins (R0). No extraprostatic extension, seminal vesicle invasion, or perineural invasion was identified. An ethical approval was taken for this publication by the Ethics committee in Medical University-Plovdiv. An informed consent was signed by the patient. The authors certify that the patient has given his consent that his images and other clinical information will be published in the journal. The patient understands that his name and initials will not be published.

The datasets generated and analyzed during the current study are available from the corresponding author on reasonable request.

## 5 In-hospital postoperative course

The postoperative course was uneventful. The patient recovered without major complications, and surgical wounds healed appropriately. After catheter removal, spontaneous voiding was achieved without urinary retention. No standardized assessment of urinary continence or erectile function was available. The patient was scheduled for routine outpatient follow-up with serial PSA measurements according to standard postoperative surveillance protocols. The serum PSA value measured on postoperative day 5 (at hospital discharge) was considered an immediate postoperative measurement and was not used for assessment of oncological outcome.

## 6 Discussion

Successful nerve-sparing radical prostatectomy depends on a detailed understanding of pelvic anatomy and precise identification of the periprostatic fascial planes. The prostate is surrounded by complex fascial structures that determine the surgical planes used during dissection. The visceral and parietal components of the endopelvic fascia form important anatomical landmarks, while the space of Retzius, puboprostatic ligaments, dorsal venous plexus, and Denonvilliers' fascia contribute to the anatomical framework encountered during radical prostatectomy (11, 12).

Preservation of the neurovascular bundles (NVBs) represents one of the most important determinants of postoperative functional recovery after radical prostatectomy. The autonomic fibers responsible for erectile function, ejaculation, and urinary control originate from the inferior hypogastric plexus and form the prostatic plexus surrounding the prostate (13). The NVBs run posterolaterally along the prostate within the periprostatic fascial layers, although their location and thickness demonstrate considerable anatomical variability, particularly near the prostatic apex (14, 15). This anatomical variability highlights the importance of careful dissection and individualized nerve-sparing strategies.

The primary objective of nerve-sparing surgery is to maximize preservation of functional structures while maintaining adequate oncological control. The degree of NVB preservation depends on the selected plane of dissection, which may range from intrafascial to interfascial or extrafascial approaches. Therefore, the optimal technique should be selected according to tumor characteristics, probability of extracapsular extension, baseline erectile function, and the patient's functional priorities.

In robot-assisted radical prostatectomy (RARP), the standard nerve-sparing technique is traditionally performed using an antegrade approach. In this technique, dissection begins with control of the prostatic pedicles, followed by progressive release of the NVBs from the prostate base toward the apex (16). Although widely accepted and reproducible, this sequence requires manipulation of vascular structures before the entire course of the neurovascular bundle has been identified. Consequently, traction or thermal injury during early pedicle control remains a potential concern, particularly in cases with challenging anatomy (17).

The retrograde nerve-sparing approach represents an alternative sequence of dissection in which the NVB is initially identified at the level of the prostatic apex and subsequently released in a distal-to-proximal direction. This approach allows the surgeon to define the anatomical course of the neurovascular structures before proceeding toward the vascular pedicles. The main theoretical advantage is improved visualization of the dissection plane and more controlled handling of the NVB during the initial stages of surgery. Although retrograde NVB dissection is not a newly introduced technique, its application in robotic surgery provides an opportunity to combine anatomical precision with the advantages of the robotic platform, including magnified three-dimensional visualization, wristed instrumentation, and improved control of delicate tissue manipulation. The educational value of this approach lies in demonstrating a reproducible surgical sequence that may assist surgeons familiar with conventional antegrade dissection who wish to expand their nerve-sparing strategies (18–20).

The retrograde NVB approach should be clearly distinguished from the Retzius-sparing technique. The Retzius-sparing approach, first described by Bocciardi et al., represents a modification of RARP aimed at preserving the anterior compartment of the pelvis, including structures involved in continence mechanisms (19, 21). Several studies have reported earlier recovery of urinary continence following Retzius-sparing RARP compared with conventional anterior approaches (22). However, Retzius-sparing surgery and retrograde NVB dissection represent different technical concepts. The former refers to preservation of the anterior pelvic space, whereas the latter describes the direction of neurovascular bundle release during nerve-sparing dissection (23, 24).

In the present case, retrograde bilateral nerve-sparing RARP was performed using the da Vinci X surgical system. The procedure was completed without intraoperative complications, and histopathological examination demonstrated organ-confined disease with negative surgical margins. Owing to the short follow-up period and the absence of appropriately timed postoperative PSA assessment, definitive conclusions regarding oncological control cannot yet be drawn. These results should be interpreted cautiously, as postoperative functional outcomes are influenced by multiple factors, including patient age, baseline function, tumor characteristics, surgeon experience, and rehabilitation protocols. Previous studies have demonstrated that RARP provides favorable perioperative outcomes compared with open surgery, including reduced blood loss, shorter hospitalization, and faster postoperative recovery (25, 26). Furthermore, preservation of the NVBs has consistently been associated with improved recovery of erectile function and urinary continence. Nevertheless, current evidence does not establish definitive superiority of retrograde over antegrade nerve-sparing dissection (27, 28). Available comparative studies remain limited, with heterogeneous patient populations and variations in surgical technique and outcome assessment.

Beyond refinement of the dissection sequence itself, contemporary nerve-sparing surgery increasingly incorporates intraoperative techniques designed to optimize the balance between oncological safety and functional preservation. The recently published multicenter randomized NeuroSAFE PROOF trial demonstrated that intraoperative frozen-section–guided nerve sparing significantly improved postoperative erectile function and early urinary continence while maintaining oncological safety compared with standard robot-assisted radical prostatectomy. These findings provide high-level evidence supporting real-time intraoperative assessment of surgical margins as an adjunct to individualized nerve-sparing surgery (29). In parallel, fluorescence confocal microscopy (FCM) has emerged as a promising alternative to conventional frozen-section analysis. Prospective studies using the Histolog Scanner system have demonstrated high diagnostic accuracy for intraoperative detection of positive surgical margins, while an international Delphi consensus has recently established recommendations for standardized implementation of FCM, including risk-adapted patient selection and integration into robotic surgical workflows (30, 31). Although these technologies were not available in the present case, they represent important future directions for tailoring nerve-sparing strategies by facilitating more confident preservation of the neurovascular bundles without compromising oncological principles.

This report has several limitations. First, it represents a single-case experience and therefore cannot determine whether the retrograde nerve-sparing approach provides superior functional or oncological outcomes compared with conventional techniques. Second, the decision to preserve the neurovascular bundles was made intraoperatively based on anatomical findings and oncological considerations. Detailed preoperative biopsy characteristics and multiparametric MRI findings, including PI-RADS classification, were unavailable in the reviewed medical documentation. These parameters are important for preoperative risk stratification and selection of the optimal nerve-sparing strategy; therefore, the rationale for nerve preservation in this patient should be interpreted within this limitation. Third, validated functional outcome measures, including preoperative and postoperative IIEF-5 and ICIQ questionnaires, were not collected. Consequently, conclusions regarding preservation of erectile function and patient-reported continence outcomes cannot be drawn from this case. Finally, advanced intraoperative margin assessment techniques, such as NeuroSAFE-guided frozen-section analysis and fluorescence confocal microscopy, were not available and therefore could not be incorporated into intraoperative decision-making. Accordingly, the present report should be regarded as a technical description demonstrating the feasibility and practical application of the retrograde nerve-sparing approach rather than evidence of its superiority over conventional techniques.

Despite these limitations, this case illustrates that retrograde nerve-sparing robot-assisted radical prostatectomy is a feasible surgical strategy in appropriately selected patients when performed by surgeons experienced in robotic pelvic surgery. Detailed anatomical knowledge, individualized surgical planning, and adherence to oncological principles remain essential for balancing cancer control with functional preservation. Further prospective comparative studies are required to define the role of retrograde nerve-sparing dissection in contemporary robot-assisted radical prostatectomy.

### 6.1 Patient perspective

The patient expressed satisfaction with the surgical outcome and reported minimal postoperative discomfort. He was particularly pleased with the rapid recovery and early return to normal daily activities. Overall, he considered the treatment experience positive and expressed confidence in the chosen surgical approach.

## 7 Conclusion

Retrograde nerve-sparing robot-assisted radical prostatectomy represents a feasible alternative dissection strategy that may facilitate precise identification and preservation of the neurovascular bundles in appropriately selected patients. This case illustrates the practical application of the retrograde approach, emphasizing its anatomical rationale and technical considerations rather than demonstrating superiority over conventional antegrade nerve sparing. Careful patient selection, sound anatomical knowledge, and adherence to oncological principles remain essential for balancing cancer control with functional preservation. Further prospective comparative studies are required to clarify the role of retrograde nerve-sparing dissection in contemporary robot-assisted radical prostatectomy.

## Data Availability

The original contributions presented in the study are included in the article/Supplementary Material, further inquiries can be directed to the corresponding author.
